# Unlocking Pragmatic Changes in Typically Aging Elderly: An In‐Depth Analysis

**DOI:** 10.1002/alz.089642

**Published:** 2025-01-09

**Authors:** Preetie Shetty Akkunje, Sasi kumar Archa, Prasanna Suresh Hegde, Michael H Thaut

**Affiliations:** ^1^ Department of Audiology and Speech Language Pathology, Kasturba Medical College Mangalore, Manipal Academy of Higher Education, Manipal, Karnataka India; ^2^ Naseema Institute of Speech and Hearing, Bangalore, Karnataka India; ^3^ Sukino Inpatient Rehabilitation Centre, Bangalore, Karnataka India; ^4^ Department of Deglutology and Speech Language Pathology, HCG Hospital, Bangalore, Karnataka India; ^5^ Music and Health Science Research Collaboratory, University of Toronto, ON Canada

## Abstract

**Background:**

The dual‐cyclical relationship between language and cognition, encapsulated in linguistic relativity, underscores the reciprocal influence of thoughts on communication and vice‐versa. This study explores the intricate changes in pragmatics, a fundamental aspect of human communication, during the aging process, considering changes in sensory abilities, cognition, and language.

**Method:**

Sixty participants, aged ≥50 years with a minimum of five years of formal education, were included, excluding those with neurological or psychological illnesses. Participants were categorized based on Montreal Cognitive Assessment (<25 & ≥25 MOCA scores), and their demographic details, language use, and educational and occupational history were recorded. Pragmatic evaluations included 1) functional communication profile analysis (sensory, receptive language, expressive language, pragmatic language and non‐oral communication), 2) pragmatic language evaluation (verbal aspects, paralinguistic aspects, nonverbal aspects), 3) spontaneous speech evaluation using Western Aphasia Battery, 4) discourse production assessment (macrostructures–main concept analysis, correct information units, global coherence and microstructures–T unit), and 5) hearing evaluation (pure tone audiometry, speech audiometry). Statistical analysis employed SPSS version 29.0.10, utilizing descriptive statistics and Likelihood ratio or Chi‐square tests.

**Results:**

Participants had a mean age of 66.5±9.9 years, with 58.3% females and 41.7% males. Educational distribution included 31.7% with ≤10th standard, 13.3% with 12th standard, 46.7% with a Bachelor’s degree, 6.7% with a Master’s degree, and 1.7% with a Ph.D. Regarding language proficiency, 41.7% were proficient in one language, 53.3% in two languages, and 5% in three languages. Significant relationships between pragmatic characteristics were identified among participant groups, supporting existing literature linking cognitive‐linguistic decline to aging. Higher MOCA scores correlated with superior speech discrimination and communication, while lower scores were associated with hearing loss. Likelihood Ratio Tests indicated significant associations between higher MOCA scores (≥25) and clear, specific, stimulus‐related pragmatic communication, while scores below 25 were linked to pragmatic difficulties.

**Conclusion:**

The study highlights the robust connection between pragmatics, cognitive function, and hearing abilities in the elderly. It offers insights into communication disorders, advocating for integrated rehabilitation programs for individuals with cognitive decline and hearing loss. Additionally, it reveals the intricate relationship between education, cognitive abilities, and pragmatic skills, guiding clinical practice and intervention strategies.

